# Causes of Cholera

**Published:** 1874-04

**Authors:** 


### CAUSES OF CHOLERA.

Conditions which excite cholera, when the disease is prevalent in a community, would not do so otherwise. Wherever cholera prevails the things which excite it—which bring it on—ought to be extensively known, with a view to guard against them—to avoid what would be likely to bring on an attack. Among these causes, all avoidable, are—

1. Places where fermenting human excrement is present.
2. A moist, warm condition of the air, causing decay of animal or vegetable substances.
3. Low, moist ground.

4. Filthy gutters, yards and cellars.
5. Bad water.
6. Food that is not nutritious or has been kept too long.
7. Bodily fatigue.
8. Low spirits.
9. Exposure to changes of weather—as cold nights after hot days.
10. Scanty supply of nourishing food.
11. Bad air.
12. Excesses—whether in eating, drinking or licentiousness.

In other words, when cholera prevails in any community, live plainly; take nourishing food at regular intervals; make no great change in any habit of life; avoid fatigue and harassment of body and mind, and cultivate a hopeful and cheerful disposition; get abundant sleep; do everything deliberately; and do all that is possible to maintain an equable condition of body and mind, with scrupulous cleanliness everywhere.

